# Caspase-8 and Caspase-9 Functioned Differently at Different Stages of the Cyclic Stretch-Induced Apoptosis in Human Periodontal Ligament Cells

**DOI:** 10.1371/journal.pone.0168268

**Published:** 2016-12-12

**Authors:** Yaqin Wu, Dan Zhao, Jiabao Zhuang, Fuqiang Zhang, Chun Xu

**Affiliations:** 1 Department of Prosthodontics, Ninth People’s Hospital, Shanghai Jiao Tong University School of Medicine, Shanghai, China; 2 Shanghai Key Laboratory of Stomatology & Shanghai Research Institute of Stomatology, Shanghai, China; University of PECS Medical School, HUNGARY

## Abstract

**Background:**

Human periodontal ligament (PDL) cells underwent apoptosis after mechanical stretch loading. However, the exact signalling pathway remains unknown. This study aimed to elucidate how the apoptotic caspases functioned in the cyclic stretch-induced apoptosis in human PDL cells.

**Materials and Methods:**

In the present study, 20% cyclic stretch was selected to load the cells for 6 or 24 h. The following parameters were analyzed: apoptotic rates, the protein levels of caspase-3, -7, -8 and -9 and the activities of caspase-8 and -9. Subsequently, the influences of caspase-8 and caspase-9 inhibitors on the apoptotic rate and the protein level of the activated caspase-3 were assessed as well.

**Results:**

The apoptotic rates increased in response to cyclic stretch, but the cells entered different apoptotic stages after 6 and 24 h stretches. Caspase-3, -7, -8 and -9 were all activated after stretch loading. The stretch-induced apoptosis and the protein level of the activated caspase-3 were inhibited after inhibiting both caspase-8 and caspase-9 in both 6 and 24 h stretched cells and after inhibiting caspase-9 in 24 h stretched cells.

**Conclusion:**

Caspase-8 and -9 functioned differently at different apoptotic stages in human PDL cells after cyclic stretch.

## Introduction

Periodontal ligament (PDL) is a fibrous structure interfacing the tooth with its surrounding bone [1]. PDL cells sensitively perceive mechanical stimuli from normal mastication, occlusal trauma or orthodontic forces [2]. There are growing evidences showing that the maintenance of periodontal health requires for physiological mechanical stimulations [3], while aberrant mechanical stimulations can be associated with pathological changes and cell death in periodontal tissue [4].

Apoptosis has been defined as a type of programmed cell death [2, 5], and can be conducted in two classic signalling pathways: the extrinsic pathway and the intrinsic pathway [6]. The extrinsic pathway is a caspase-dependent pathway and is extensively used to describe the process of apoptosis induced by extracellular stimulations which are recognized and propagated by the specific membrane receptors. The intrinsic pathway involves a mitochondrion-centered control mechanism via either a caspase-dependent pathway or a caspase-independent pathway, and can be triggered by multiple intracellular stimulations [7]. The extrinsic pathway is mediated by caspase-8 while the intrinsic pathway can be initiated through caspase-9, and both pathways trigger apoptosis through the cleavage of the downstream executioner proteins, caspase-3 and -7 [8].

Mechanical stress can lead to a series of biological responses in periodontal ligament, including alterations of cellular behavior, gene expression and protein production [9, 10]. Several studies revealed cell apoptosis in PDL under orthodontic forces in animal experiments *in vivo* [11, 12]. In our previous studies, cell death via apoptosis was also found in human PDL cells in response to cyclic stretching [13, 14]. The early and the late apoptosis were induced notably after 6 and 24 h 20% cyclic stretch loading, respectively [13, 14]. Wang et al. also reported that apoptosis of certain extent was induced by 20% cyclic stretching force, with a higher apoptotic rate after 24 h stretch than that after 6 h stretch [15]. Meanwhile, our recent study uncovered that apoptosis in human PDL cells after cyclic stretch occurred through the activation of caspase-3 via caspase-9 [14]. The differential expression of some force-sensing genes with regard to apoptosis, such as *FAS* and so on, was also detected [16].

Results of previous researches indicated that different duration of cyclic stretches induced apoptosis of different stages in human PDL cells, and the stretch-induced apoptosis involved a complicated signalling pathway. However, the precise mechanism underlying the stretch-induced apoptotic pathway in human PDL cells is unknown. The present study aimed to provide novel insight into the roles of caspase-8 and caspase-9 at different stages of the stretch-induced apoptosis in *in vitro* cultured human PDL cells. We hope the findings in this study will help us better understand the mechanism of the force-driven apoptosis in PDL cells.

## Materials and Methods

### Cell culture

The experimental protocol is approved by the Ethics Committee of Ninth People’s Hospital, Shanghai Jiao Tong University School of Medicine. The relevant Judgement's reference number: [2008]17. Human PDL tissues were harvested from extracted premolars of teenagers (11–13 years old) undergoing orthodontic treatments. Written informed consents were obtained from their parents. Human PDL cells were prepared *in vitro* according to the protocols described previously [17, 18]. High glucose DMEM (HyClone, Logan, Utah, USA), FBS (Gibco, Carlsbad, CA, USA) and antibiotics (HyClone) were applied to cell culture. Pieces of human PDL tissues were incubated in the DMEM supplemented with 20% (v/v) FBS and five-fold reinforced antibiotics (500 U/mL penicillin and 500 μg/mL streptomycin). When cells grew out from the tissue pieces and reached 80%-90% confluence, they were passaged and cultured in DMEM with 10% (v/v) FBS and antibiotics (100 U/mL penicillin and 100 μg/mL streptomycin). Human PDL cells were cultured in a 37°C humidified incubator with 5% CO_2_ in air. Cells at passage 4 to 6 were used for the subsequent experiments.

### Loading of cyclic stretch

The CSU was applied to stretch the human PDL cells, as described in our recent studies previously [13, 14, 16–18]. The CSU is comprised of three components, a strainer, a controller and a PC. Cells were passaged onto a cell culture dish (diameter 60 mm), with an elastic silicon rubber membrane (Q7-4750, Dow Corning Co., Midland, MI, USA) in the bottom. The flexible membrane was deformed by the spherical cap moving up and down repeatedly, and therefore, the attached cells were stretched. The controller and the PC controlled all changes in the stretch strain and the movement of the spherical cap.

The human PDL cells were passaged at a concentration of 1.5 ×10^6^ cells per dish onto the flexible-bottomed cell culture dishes. When the cells achieved 70%-80% confluence, they were subjected to the cyclic stretch with 20% strain for 6 or 24 h at a frequency of 6 cycles/min (5 s stretch and 5 s relaxation). PDL cells cultured in a similar condition but without stretch loading served as non-stretching controls. Yamaguchi et al. reported that stretching strain less than 24% is appropriate to mimic the strain confronted by human PDL cells *in vivo* [19]. Additionally, our recent studies reported that the early and the late apoptosis were apparently enhanced in response to 6 and 24 h 20% stretch strains, respectively [13, 14]. Therefore, the cultured cells were stretched by cyclic stretch of 20% strain for 6 or 24 h in the present study. The loading frequency of 6 cycles/min (5 s stretch and 5 s relaxation) was the same as that in our previous studies [18]. For experiments of apoptosis analysis, caspases activity analysis and Western Blot analysis, three independent treatments were carried out in every group.

### Morphological observation

At the end of the stretch loading, the cells were observed under an inverted phase-contrast light microscope (Leica DMRIRB, Bensheim, Germany) to determine the morphological changes after cyclic stretch.

### Analysis of apoptosis by flow cytometry

Apoptotic rate was detected by annexin V/PI double staining method using an FITC Annexin V Apoptosis Detection Kit (Becton Dickinson, San Jose, CA, USA), according to the manufacturer’s instruction. The supernatant and the cells of the sample were collected and were centrifuged in 1000 r/min for 5 min to get the sediment. After re-suspension in Binding Buffer, the cells were stained with FITC Annexin V and PI for 15 min at room temperature in darkness. All samples were tested by using a BD FACSCalibur (Becton Dickinson) within 1 h. For the inhibitory experiment, the cells were incubated with the specific inhibitor of caspase-8 (10 μM) (Z-IETD-FMK, Biovision Research Products, Mountain View, CA, USA) or the specific inhibitor of caspase-9 (10 μM) (Z-LEHD-FMK, Biovision Research Products) or both of them for 1 h at 37°C before stretch loading. In the present study, Annexin V positive and PI negative stained (Annexin V+/PI-) cells were recorded to be at the early apoptotic stage, whereas Annexin V and PI positive stained (Annexin V+/PI+) cells were recorded to be at the late apoptotic stage.

### Caspase-8 and caspase-9 activity assay

The activity of caspase-8 and caspase-9 was assessed, respectively, with the corresponding Colorimetric Assay Kit (Biovision Research Products) according to the manufacturer’s instruction. The collected cells were lysed for 10 min on ice to get the protein. The BCA method was used to measure the protein concentration with the Enhanced BCA Protein Assay Kit (Beyotime, Shanghai, China) according to the manufacturer’s instruction. 100μg protein in 50 μl Cell Lysis Buffer was added with 50 μl 2× Reaction Buffer containing 10 mM DTT and 4 mM labeled substrate (caspase-8:IETD-pNA; caspase-9:LETD-pNA). The suspension was incubated at 37°C for 1 h. The absorbance of each sample was read at 400 nm in a 96-well plate reader (ELx800, Biotec, Vermont, USA).

### Western Blot analysis

Total protein was obtained from the collected cells by using the RIPA buffer (Sigma). Like the aforementioned method, the protein concentration was also assayed by using the BCA method with the Enhanced BCA Protein Assay Kit (Beyotime). Equal amount of protein from each sample was separated by 12% SDS-PAGE (Beyotime), and then the protein was transferred to a 0.22μm PVDF membrane (KeyGEN, Nanjing, China). Following being blocked, the membrane was incubated with the specific primary antibody, including monoclonal Caspase-3 (8G10) Rabbit mAb (1:500), monoclonal Caspase-7 (D2Q3L) Rabbit mAb (1:1000), monoclonal Caspase-8 (1C12) Mouse mAb (1:1000) and polyclonal Caspase-9 Antibody (Human Specific) (1:1000) (Cell Signaling Technology, Danvers, MA, USA), at 4°C overnight. Next, the membrane was washed for three times with PBS containing 0.1% Tween 20 (PBST) (5 min each) and was incubated with the secondary antibody (anti-mouse, 1:2000; anti-rabbit, 1:5000; KangChen, Shanghai, China) at room temperature for 1 h. After being washed with PBST again for three times (15 min each), the membrane was visualized using an ECL reagent (SuperSignal West Femto Substrate, Thermo Scientific, Waltham, MA, USA) to get the blot. Image-Pro Plus 6.0 (MediaCybernetics, Inc., Bethesda, MD, USA) was used to analyze the protein level. For the inhibitory experiment, the human PDL cells were treated with the specific inhibitor of caspase-8 (10 μM) (Z-IETD-FMK, Biovision Research Products) or the specific inhibitor of caspase-9 (10 μM) (Z-LEHD-FMK, Biovision Research Products) or both of them for 1 h at 37°C before stretch loading.

### Statistical analysis

The results were expressed as means ± standard deviation (n = 3) and were statistically analyzed with SPSS software, version 19.0 (IBM Corporation, Armonk, NY, USA). The differences among groups were analyzed by using one-way analysis of variance (ANOVA) followed with Student-New-Keul’s Post-hoc test and P<0.05 was set as the statistical significance level.

## Results

### Cyclic stretch changed the morphology and the alignment of human PDL cells

**Fig 1:**
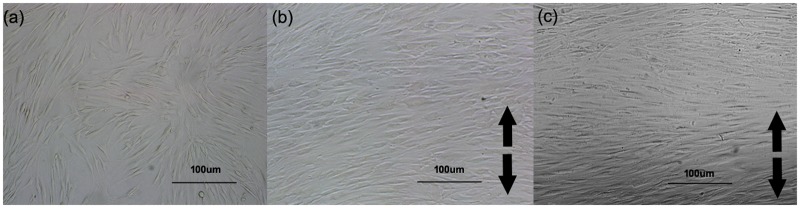
Cyclic stretch changed the morphology of the human PDL cells. (a) non-stretching control. Cells aligned multidirectionally. (b) 6 h stretched cells. (c) 24 h stretched cells. (b-c) Cells were elongated and prone to be parallel to each other, with their long axis aligned perpendicularly to the force vector. 24 h stretched cells were altered more significantly. Black arrow showed the stretching direction.

Remarkable alterations of the morphology and the alignment of human PDL cells were observed under the light microscope after 20% stretch strain loading. In response to stretching, cells were elongated and prone to be paralleled to each other, with their long axis aligned perpendicularly to the stretch force vector. The 24 h stretched cells were altered more significantly. (Fig 1) This phenomenon was consistent with previous reports [13, 17].

### Cyclic stretch for 6 and 24 h induced apoptosis of different stages

**Fig 2:**
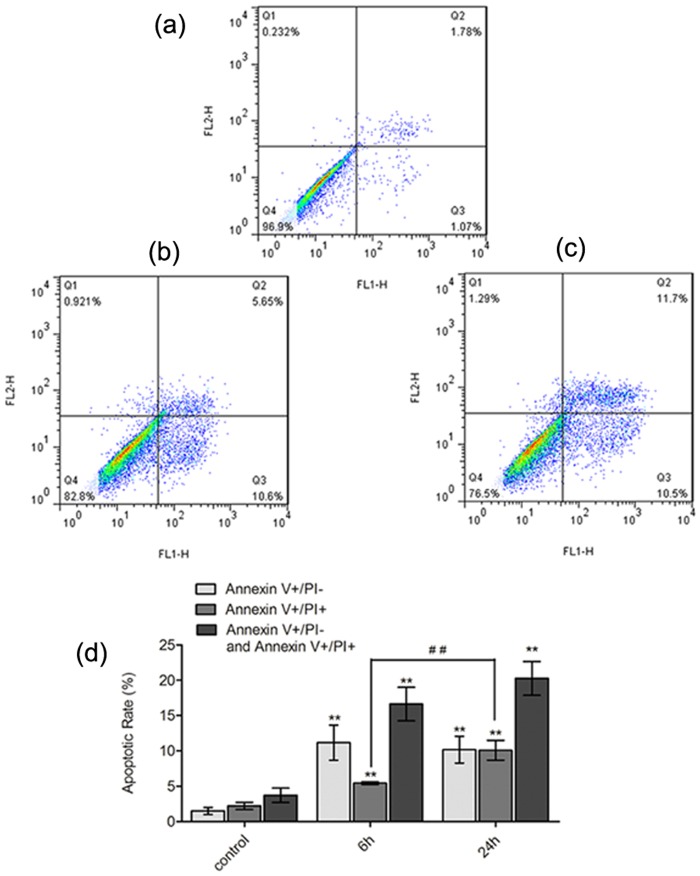
Cyclic stretch for 6 and 24 h induced apoptosis of different stages. (a-c) Dot plots of Annexin V and PI staining were obtained from flow cytometry. The lower right quadrant (Annexin V+/PI-) reflects cells at early stage of apoptosis, while the upper right quadrant (Annexin V+/PI+) indicates cells at late stage of apoptosis. (d) Apoptotic rates in each group were analyzed. (a) non-stretching control. (b) 6 h stretched cells. (c) 24 h stretched cells. (d) The apoptotic rates (including the early and the late apoptosis) increased in the 6 and 24 h stretched groups (P<0.01 VS non-stretching control). The apoptotic rates in the 6 h stretched cells increased mainly due to the up-regulation of the early apoptosis (P<0.01 VS non-stretching control), and 24 h stretch further raised the late apoptotic rate apparently (P<0.01 VS 6 h stretch). Bars represent standard deviations (n = 3). **: *P*<0.01 VS non-stretching control; ##: P<0.01 VS 6 h stretch.

Comparing with that of the non-stretching control, the apoptotic rate (including the early and the late apoptosis) increased remarkably after 6 h stretch loading, mainly due to the up-regulation of the early apoptosis. Cyclic stretch for 24 h also raised the apoptotic rate (including the early and the late apoptosis) apparently, but with more cells entering the late stage of apoptosis. (Fig 2)

### Cyclic stretch increased the protein levels of the activated caspase-3, -7, -8 and -9

**Fig 3:**
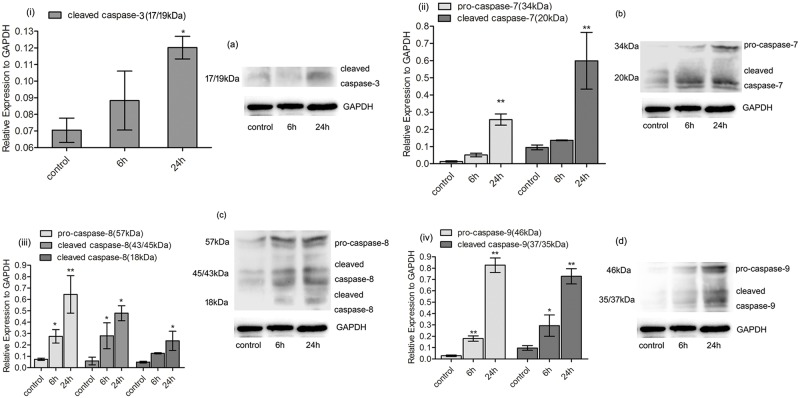
Cyclic stretch induced the expression of activated caspase-3, -7, -8 and -9. Western Blot results for the protein level of caspase-3, -7, -8 and -9. (i-iv) Mean expression levels of the proteins normalized to GAPDH, from three independent experiments, in response to 6 and 24 h stretch loading. (a-d) Representative protein bands of Western Blot experiments. The protein level of the cleaved caspase-3 (P<0.05) and the cleaved caspase-7 (P<0.01) and up-regulation of the pro-caspase-7 (P<0.01) were detected after 24 h stretch. The up-regulation of the cleaved caspase-8 (P<0.05), the cleaved caspase-9 (6 h stretch: P<0.05; 24 h stretch: P<0.01), the pro-caspase-8 (6 h stretch: P<0.05; 24 h stretch: P<0.01) and the pro-caspase-9 (P<0.01) were detected in both 6 and 24 h stretched cells. Bars represent standard deviations (n = 3). **: P<0.01 VS non-stretching control; *: P<0.05 VS non-stretching control.

The protein levels of the cleaved caspase-3, -7 and the pro-caspase-7 increased after 24 h stretch loading, comparing with those of the non-stretching control. Furthermore, the up-regulation of the cleaved caspase-8 and caspase-9 in response to both 6 and 24 h stretch loading was detected. The protein levels of the pro-caspase-8 and the pro-caspase-9 in response to both 6 and 24 h stretch loading were also up-regulated, comparing with those of the non-stretching control. (Fig 3)

### Cyclic stretch increased the activities of caspase-8 and caspase-9

**Fig 4:**
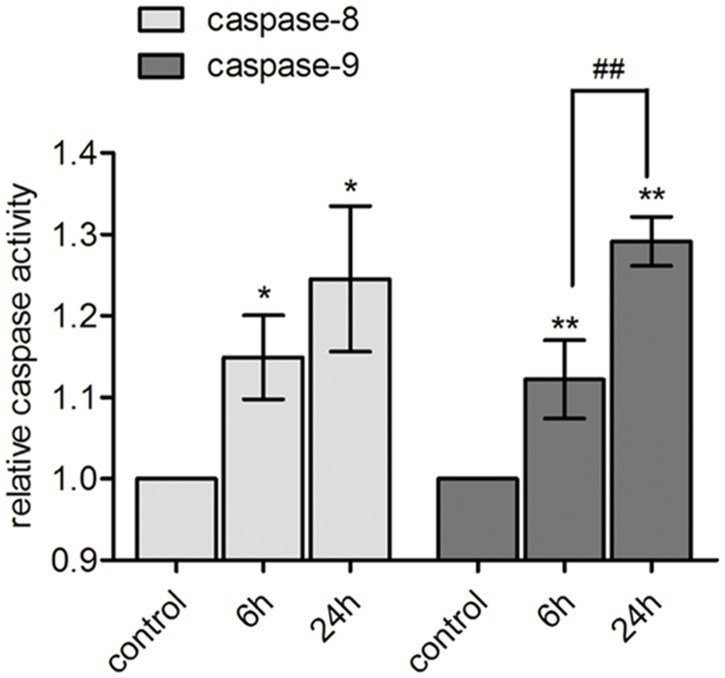
Cyclic stretch increased the activity of caspase-8 and caspase-9. The results of activity analysis of caspase-8 and -9. Compared with the non-stretching control, the caspase-8 (P<0.05) and the caspase-9 (P<0.01) activities in human PDL cells in response to 20% cyclic stretch for 6 or 24 h increased significantly. Cyclic stretch for 24 h further increased the activity of caspase-9, comparing with 6 h stretch (P<0.01). Bars represent standard deviations (n = 3). **: *P*<0.01 VS non-stretching control; *: P<0.05 VS non-stretching control; ##: P<0.01 VS 6 h stretch.

Comparing with the non-stretching control, 6 and 24 h stretches of 20% strain raised the activities of caspase-8 and caspase-9. Cyclic stretch for 24 h further increased the activity of caspase-9, comparing with 6 h stretch. (Fig 4) (S1 Table)

### Inhibition of both caspase-8 and caspase-9 suppressed the 6 h stretch-induced apoptosis

**Fig 5:**
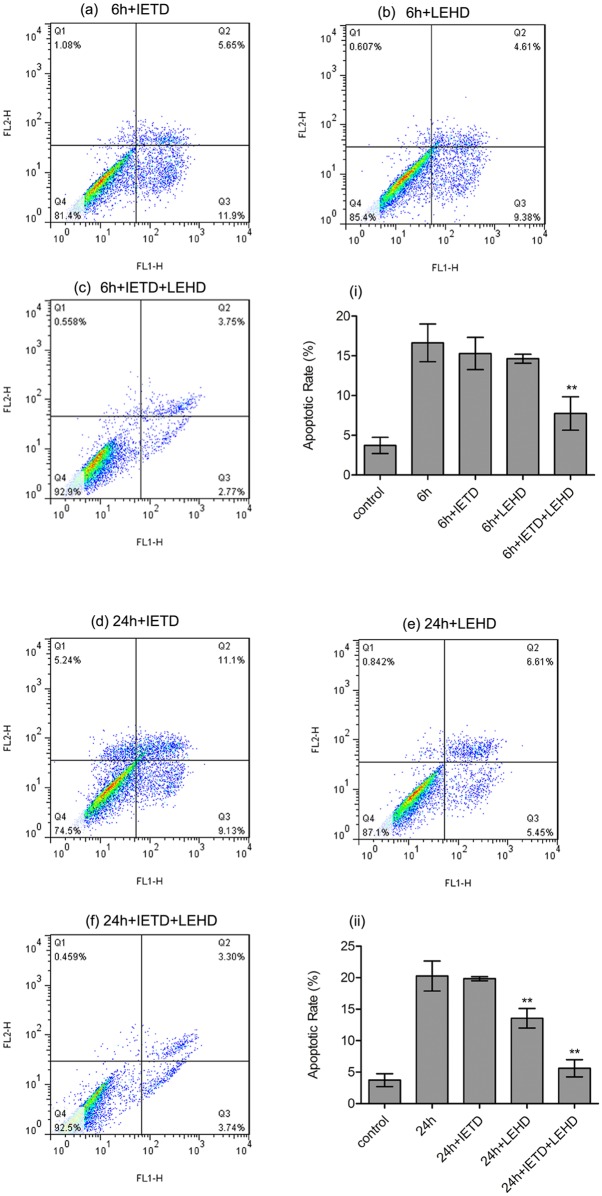
Inhibition of caspase-8 and caspase-9 suppressed the apoptotic rate. The results of apoptotic analysis after inhibiting either caspase-8 or caspase-9 or both of them. (a-f) Dot plots of Annexin V and PI staining. (i, ii) Apoptotic rates in each group were calculated. In 6 h stretched cells, the apoptotic rates (including the early and the late apoptosis) were identified no statistical difference by using either caspase-8 inhibitor or caspase-9 inhibitor (P>0.05). However, inhibition of both caspase-8 and caspase-9 significantly reduced the apoptotic rates in 6 h stretched cells (P<0.01). In 24 h stretched cells, the apoptotic rate was significantly down-regulated by using caspase-9 inhibitor (P<0.01), while showed no change by using caspase-8 inhibitor (P>0.05). Inhibition of both caspase-8 and caspase-9 also significantly reduced the apoptotic rate in 24 h stretched cells (P<0.01). **: P<0.01 VS non-inhibiting cells.

**Fig 6:**
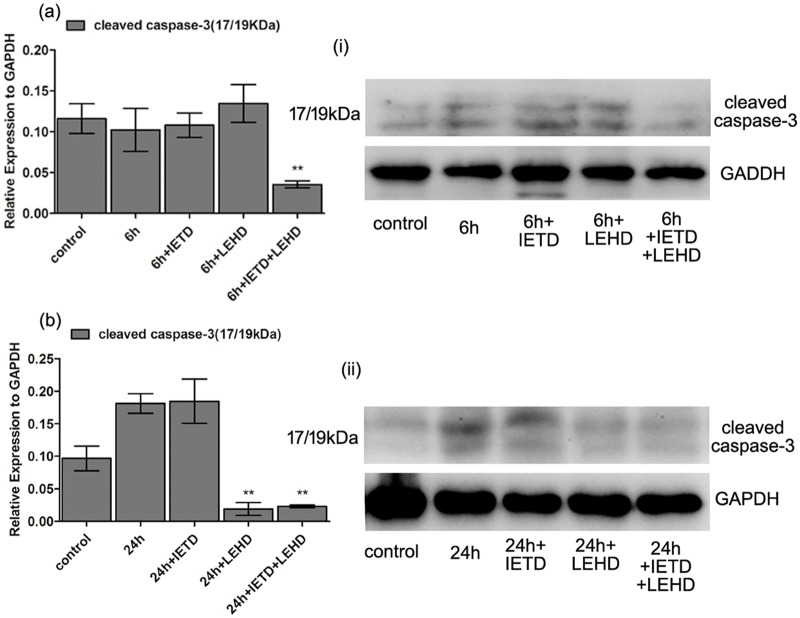
Inhibition of caspase-8 and caspase-9 suppressed the protein level of the cleaved caspase-3. Western Blot results after inhibiting either caspase-8 or caspase-9 or both of them. (i, ii) Representative protein bands of Western Blot experiments. (a, b) Mean expression levels of the proteins normalized to GAPDH, from three independent experiments. In 6 h stretched cells, the activation of caspase-3 were identified no statistical difference by using either caspase-8 inhibitor or caspase-9 inhibitor (P>0.05). However, inhibition of both caspase-8 and caspase-9 significantly reduced the protein level of the cleaved caspase-3 in 6 h stretched cells (P<0.01). In the 24 h stretched cells, caspase-9 inhibitor alone reduced the protein level of the cleaved caspase-3 (P<0.01), while only using caspase-8 inhibitor had no inhibitory effect on the protein level of the cleaved caspase-3 (P>0.05). Inhibition of both caspase-8 and caspase-9 also significantly reduced the protein level of the cleaved caspase-3 in 24 h stretched cells (P<0.01). Bars represent standard deviations (n = 3). **: P<0.01 VS non-inhibiting cells.

In the 6 h stretched cells, neither caspase-8 inhibitor (Z-IETD-FMK) nor caspase-9 inhibitor (Z-LEHD-FMK) decreased the stretch-induced apoptotic rate or the protein level of the cleaved caspase-3. While the apoptotic rate and the protein level of the cleaved caspase-3 were significantly down-regulated with the use of both caspase-8 inhibitor (Z-IETD-FMK) and caspase-9 inhibitor (Z-LEHD-FMK) in 6 h stretched cells, compared with those in the non-inhibiting stretched cells. (Figs 5 and 6)

### Inhibition of caspase-9 but not caspase-8 suppressed the 24 h stretch-induced apoptosis

Caspase-8 inhibitor didn’t show any inhibitory effect on the 24 h stretch-induced apoptosis or the activation of caspase-3. However, the apoptotic rate after 24 h stretch loading significantly decreased with the use of caspase-9 inhibitor. What’s more, comparing with those in the non-inhibiting stretched cells, the protein levels of the cleaved caspase-3 were reduced by caspase-9 inhibitor in the 24 h stretched cells. (Figs 5 and 6)

## Discussion

PDL plays an important role in equilibrating the force from the tooth to the bone and in maintaining periodontal homeostasis. PDL cells sensitively perceive mechanical stress and are actively involved in the biological effects of the force on periodontium. It have been reported that PDL cells disappeared at the pressure side of PDL via apoptosis during orthodontic tooth movement [11]. Whereas, it was suggested that apoptosis was also occurred at the tension side of PDL [12]. In our previous studies, it was found that human PDL cells underwent apoptosis under *in vitro* cyclic stretch [13, 14]. Nevertheless, there is still a dearth of literature about the signalling pathway of the force-driven apoptosis in PDL cells.

Under the light microscope, human PDL cells were found elongated and assumed a spindle shape after cyclic stretch loading. Besides, the cells inclined parallel to each other and aligned with their long axes perpendicular to the stretching direction. The similar phenomenon has been previously reported [9, 10] and was believed to be important for PDL cells in maintaining the periodontal tissue structure [20].

Annexin V/PI double staining method has been widely applied to measure the apoptotic rate and distinguish the early apoptosis from the late apoptosis. In our previous studies, it was reported that the early apoptotic rate reached a peak after 6 h cyclic stretching while the late apoptotic rate notably increased when the stretching time increased to 24 h [13, 14]. Consistent with these previous results, most apoptotic cells were detected to be in the early stage after 6 h stretch, and 24 h stretch loading further raised the late apoptotic rate apparently in the present study. These results suggest that the stages of apoptosis changed with the stretching time increased in human PDL cells. Therefore, different apoptotic pathways may be involved after 6 and 24 h stretches respectively.

Caspase-3 and caspase-7, known as the executioner caspases [21], exhibit the highest similarity in structure and function among all caspases members and act as an indispensable part in the executing stage of the apoptosis [22]. Immunohistochemical detection of active caspase-3 and active caspase-7 has been reported in human gingival tissue from patients with chronic periodontitis [23, 24, 25]. As caspase-3 and caspase-7 function in their proteolytically cleaved forms, the protein levels of the cleaved caspase-3 and caspase-7 were analyzed here. Results showed up-regulation of the cleaved caspase-3, -7 and the pro-caspase-7 after 24 h stretch, confirming the involvement of caspase-3 and caspase-7 in the stretch-induced apoptosis of human PDL cells. Whereas, there was no statistical alteration in the protein levels of the cleaved caspase-3, -7 and the pro-caspase-7 in response to 6 h stretch. Our previous research also demonstrated that, though there was no significant up-regulation by 6 h stretch, the activity of caspase-3 gradually increased with the stretch duration elongated and reached a peak after 24 h stretch [14]. Taking into account our previous findings that some anti-apoptotic genes such as *BAG3*, *BIRC3* and *BIRC6* were up-regulated by 6 h stretch [16], it’s likely that the unaltered protein levels of caspase-3 and caspase-7 after 6 h stretch were due to a self-protective mechanism of the cells at the initial stage of stretch loading.

In the present study, 6 and 24 h stretches induced the activation of both caspase-8 and caspase-9. Inhibiting caspase-8 or caspase-9 alone didn’t significantly reduce the apoptotic rate in the 6 h stretched cells, and didn’t alter the protein level of the cleaved caspase-3 as well, while inhibiting both caspase-8 and caspase-9 significantly suppressed the 6 h stretch-induced apoptosis and the protein level of the cleaved caspase-3. In the 24 h stretched cells, the inhibition of caspase-9 alone or both caspase-8 and caspase-9 significantly inhibited the stretch-induced apoptotic rate and the protein level of the cleaved caspase-3. These results were in accordance to our previous results of real-time PCR array analysis, which showed that the gene expression of caspase-8 and caspase-9 was up-regulated in response to 6 and 24 h cyclic stretches respectively [16]. Our previous research also demonstrated that 24 h cyclic stretch induced notable apoptosis and up-regulated the activity of caspase-3 in human PDL cells, which could be reduced by the inhibition of caspase-9 [14], suggesting the involvement of the intrinsic pathway in the stretch-induced apoptosis. On the other hand, up-regulation of the expression of some pro-apoptotic genes, including *FAS* and *TNFRSF10B*, has been found after 6 and 24 h cyclic stretch loading [16]. *Fas* and *TNFRSF10B* were reported participating in recruiting and activating caspase-8 and ultimately leading to the apoptosis [26]. Taking together these previous and present results, it is reasonable to suggest that both the extrinsic and intrinsic pathways might have been involved in the cyclic stretch-induced apoptosis in PDL cells, but functioned differently in the apoptosis induced by 6 and 24 h stretches respectively. As to *in vivo* situation, Tu et al. and Leone et al. reported the results of immunohistochemistry analysis suggesting the presence of caspase-9 in gingivae from SD rats and from human tissue, respectively [27, 28]. With respect to caspase-8, though there were no published studies showing in situ labelling for caspase-8 in periodontal tissue, the caspase-8 immunohistochemically positive fibroblast-like cells have been observed in human implant interface membrane [29]. Actually, there is a large body of evidence demonstrating the existence of caspase-3, -7, -8 and -9 in human tissue. However, how these caspases act in apoptosis *in vivo* remains to be elucidated.

It has been recognized that extrinsic apoptosis induced by extracellular stimuli can be initiated by the formation of DISC, which is a platform activating caspase-8 [30]. The activated caspase-8 can trigger the activation of caspase-3 and initiate apoptosis. As to the intrinsic pathway, multiple intracellular stimulations, such as ROS and calcium, can cause MOMP in cells. Thereby, mitochondria are triggered to release cytochrome c subsequently. Cytochrome c and Apaf-1 are combined together to form the apoptosome, which is quite necessary in activating caspase-9. The cleavage of caspase-9 enables the proteolytic maturation of caspase-3 and, consequently, the executive phase of cell apoptosis was induced [31, 32]. In some types of cells, the activation of caspase-8 directly cleaves caspase-3 irrespective of mitochondria. However, in some other types of cells, the activated caspase-8 cleaves Bid (a Bcl-2 family member) into tBid [33, 34]. Subsequently, the fragment tBid induces MOMP in cells and thereby the activation of caspase-9 is involved [33,34]. It was reported that the efficient and rapid activation of caspase-8 by DISC could directly activate caspase-3 regardless of mitochondria. Otherwise, when the formation of DISC was inefficient, caspase-8 would function in a mitochondrion-dependent manner via caspase-9 to initiate apoptosis [35,36].

**Fig 7:**
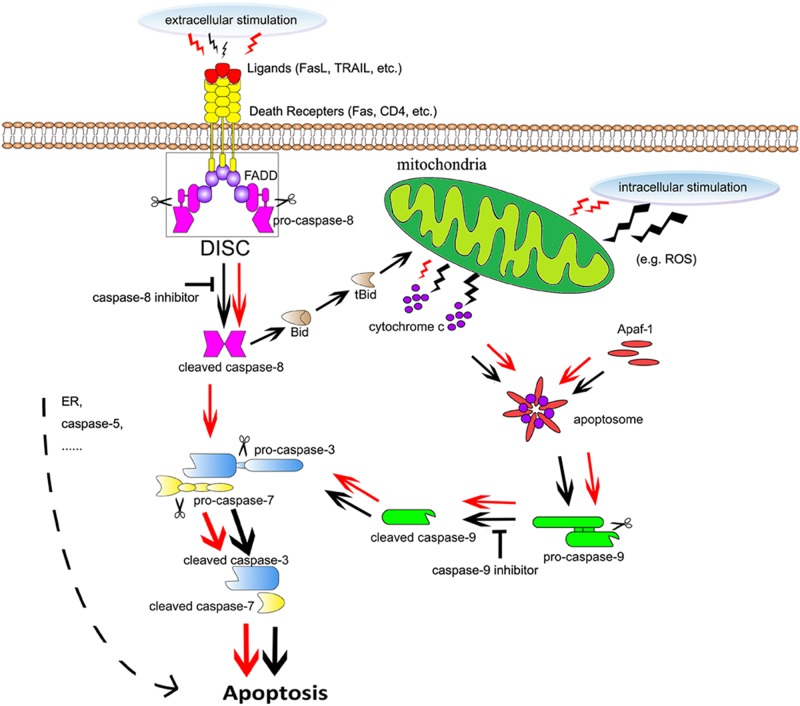
Schematic of the apoptotic pathway in the 6 and 24 h stretched cells. The red and the black arrows showed the apoptotic pathways in the 6 and 24 h stretched cells, respectively. After 6 h stretch, the extrinsic and the intrinsic pathways contributed equally to the stretch-induced apoptosis. The cleaved caspase-8 was capable of directly activating caspase-3. After 24 h stretch, the caspase-3 was activated both directly by caspase-9 through the intrinsic pathway and indirectly by caspase-8 in a mitochondrion-dependent manner via caspase-9.

Based on these considerations, a possible explanation for the findings of the present study could be proposed as follows (Fig 7). Both extrinsic and intrinsic pathways served significantly in the cyclic stretch-induced apoptosis, but acted differently at different stages. During the early stage of stretch loading (no longer than 6 h stretching) (Fig 7, red arrows), the extrinsic and the intrinsic pathways contributed equally to the stretch-induced apoptosis. The cleaved caspase-8 was capable of directly triggering the activation of caspase-3 and initiating apoptosis. So just blocking one of these two initiators (caspase-8 and -9) could not inhibit the apoptosis obviously, but blocking both of them could significantly reduce the 6 h stretch-induced apoptosis. While with the stretch duration increased to 24 h (Fig 7, black arrows), as the combination of the death receptors and its specific ligands reached saturation, the formation of DISC was inefficient and the activation of caspase-3 by caspase-8 occurred mainly in a mitochondrion-dependent manner via caspase-9. Therefore, caspase-9 inhibitor would block both extrinsic and intrinsic pathway, and caspase-8 could not counteract the effect of caspase-9 inhibitor, in the 24 h stretched cells. It would also be possible to explain why just blocking caspase-8 alone could not significantly reduce the apoptosis after 24 h stretch, because the stretch-induced apoptosis would still be carried out in the intrinsic pathway via caspase-9 in the 24 h stretched cells.

In summary, this study proposed a hypothesis that both caspase-8 and caspase-9 contributed to the cyclic stretch-induced apoptosis, but functioned differently at different stages in human PDL cells (Fig 7). Indeed, cell apoptosis is a complicated and dynamic process, involving several interconnected signalling pathways. This study did not take other factors influencing force-driven apoptosis into account and inevitably resulted in some limitations. Many reports have described the intracellular accumulation of ROS and calcium in response to mechanical forces in many types of cells [37–39] and the close relationship between ROS and calcium with the intrinsic apoptosis [40, 41]. Researchers demonstrated that the level of ROS significantly increased in PDL fibroblasts exposed to mechanical compression [38, 41]. What’s more, it has been reported that excess endoplasmic reticulum stress, induced by unfolded and malfolded proteins, mediated another apoptotic pathway [42,43]. Interestingly, our recent studies have uncovered the remarkable up-regulation of CASP5 (encoding the caspase family member, caspase-5) in response to stretch loading in human PDL cells [16]. Some studies have suggested a potential role of caspase-5 in apoptosis as well [44–46]. Therefore, whether there are other signalling pathways involved and the role of caspase-5 in the cyclic stretch-induced apoptosis are interesting questions and require for further investigation.

## Supporting Information

S1 Table Data of the caspase-8 and caspase-9 activities. (PDF)
