# Supplementary material for: Caspase-8 and Caspase-9 Functioned Differently at Different Stages of the Cyclic Stretch-Induced Apoptosis in Human Periodontal Ligament Cells
Source: PLoS One. 2016 Dec 12;11(12):e0168268. doi: 10.1371/journal.pone.0168268 (PMC5152893; doi:10.1371/journal.pone.0168268)
Supplement: S1 Table — (PDF) [file pone.0168268.s001.pdf]

Data of the caspase-8 and caspase-9 activities in human PDL cells before and after cyclic stretch.

article title Caspase-8 and Caspase-9 Functioned Differently at Different Stages of the Cyclic Stretch-Induced Apoptosis in Human Periodontal Ligament Cells

author names Yaqin Wu, Dan Zhao, Jiabao Zhuang, Fuqiang Zhang, Chun Xu

corresponding author E-mail address: imxuchun@163.com

1 Department of Prosthodontics, Ninth People’s Hospital, Shanghai Jiao Tong University School of Medicine, Shanghai, China
2 Shanghai Key Laboratory of Stomatology & Shanghai Research Institute of Stomatology, Shanghai, China

	caspase-8						caspase-9					
	activity	relative activity	activity	relative activity	activity	relative activity	activity	relative activity	activity	relative activity	activity	relative activity
control	0.0940	1.0000	0.0970	1.0000	0.0920	1.0000	0.0890	1.0000	0.0900	1.0000	0.0850	1.0000
6h	0.1040	1.1064	0.1100	1.1340	0.1110	1.2065	0.1020	1.1461	0.0960	1.1461	0.0980	1.1529
24h	0.1130	1.2021	0.1150	1.1856	0.1240	1.3478	0.1180	1.3258	0.1150	1.3258	0.1080	1.2706
